# Biodiversity Information of benthic Species at ARtificial structures – BISAR

**DOI:** 10.1038/s41597-025-04920-1

**Published:** 2025-04-10

**Authors:** Jennifer Dannheim, Paul Kloss, Jan Vanaverbeke, Ninon Mavraki, Mirta Zupan, Vanessa Spielmann, Steven Degraer, Silvana N. R. Birchenough, Urszula Janas, Emma Sheehan, Katharina Teschke, Andrew B. Gill, Zoe Hutchison, Drew A. Carey, Michael Rasser, Jolien Buyse, Babeth van der Weide, Oliver Bittner, Paul Causon, Roland Krone, Marco Faasse, Alexa Wrede, Joop W. P. Coolen

**Affiliations:** 1 Alfred Wegener Institute, Helmholtz Centre for Polar and Marine Research, Am Handelshafen 12, 27570 Bremerhaven, Germany; 2 Helmholtz Institute for Functional Marine Biodiversity at the University of Oldenburg, Ammerländer Heerstraße 231, 26129 Oldenburg, Germany; 3 Royal Belgian Institute of Natural Sciences, Operational Directorate Natural Environment (OD Nature), Marine Ecology and Management (MARECO), Vautierstraat 29, Brussels, B-1000 Belgium; 4 Wageningen Marine Research, Ankerpark 27, 1781 AG Den Helder, The Netherlands; 5 Marine Biology Research Group, Department of Biology, Ghent University, Krijgslaan 281-S8, 9000 Ghent, Belgium; 6 University of Bremen, Bibliothekstraße 1, 28359 Bremen, Germany; 7 The Centre for Environment, Fisheries and Aquaculture Science (Cefas), Pakefield Road, Lowestoft, NR33 0HT United Kingdom; 8 Environmental Resources Management (ERM) Ltd, 8 Thorpe Road, Norwich, NR1 1RY United Kingdom; 9 Department of Marine Ecology, Faculty of Oceanography and Geography, University of Gdansk, Al. Marsz. J. Pilsudskiego 46, Gdynia, 81-378 Poland; 10 School of Biological and Marine Sciences (Faculty of Science and Engineering), University of Plymouth, Plymouth, United Kingdom; 11 University of St Andrews, School of Biology, Scotland, United Kingdom; 12 INSPIRE Environmental, Newport, Rhode Island 02840 US; 13 Bureau of Ocean Energy Management, Sterling, VA 22066 US; 14 Flanders Research Institute for Agriculture, Fisheries and Food, ILVO Marine Research, Jacobsenstraat 1, 8400 Ostend, Belgium; 15 NIRAS (Group) UK, Kings Ride Court, Kings Ride, Ascot, SL5 7JR UK; 16 Reefauna, Schleusenstraße 3, 27568 Bremerhaven, Germany; 17 Eurofins Aquasense, Korringaweg 7, 4401 NT Yerseke, The Netherlands; 18 Alfred Wegener Institute, Helmholtz Centre for Polar and Marine Research, Biologische Anstalt Helgoland, Shelf Sea System Ecology, 27498 Helgoland, Germany

**Keywords:** Biodiversity, Population dynamics, Community ecology

## Abstract

Understanding the effects of artificial structures in marine landscapes is required for ecosystem-based management. Global demand for oil and gas and accelerated commitments to renewable energy development has led to the proliferation of marine artificial structures. Investigating the cumulative effects of these structures on marine ecosystems requires data on the benthic community over large geographical and long-time scales. It is imperative to share the data collected by many stakeholders in an integrated information system to benefit science, industry and policy. BISAR is the first data product containing harmonised and quality-checked international data on benthos from artificial structures in the North Sea. BISAR was compiled from environmental impact assessment studies and scientific projects (3864 samples, 890 taxa). Data derive from 34 artificial structures and surrounding soft sediments (years: 2003 to 2019). Structures include offshore wind turbines, oil and gas platforms and a research platform. Data from a geogenic reef, allow comparison of natural and artificial reef communities. We aim to host future BISAR data dynamically in the CRITTERBASE web portal.

## Background & Summary

Worldwide reduction of carbon emissions is needed to help reduce the effects of climate change. Twenty-seven member states of the European Union have committed to reduce emissions by 55% of 1990 levels by 2030^1^. To achieve this, an unprecedented installation of offshore marine renewable energy devices (wind, wave, tidal, solar) and cable networks is required^2^. To date, offshore wind energy is the largest marine renewable energy provider, currently producing globally 35 GW with an increase to 70 GW expected by 2025^3^ and a potential increase worldwide to 1000 GW expected by 2050^4^. Europe has the majority of offshore wind farms (OWFs) with a capacity of 28 GW^5^, which corresponds to 5,795 grid-connected wind turbines across 123 OWFs and 12 countries^5^.

Marine biodiversity and their associated ecosystems are increasingly being affected by anthropogenic pressures, such as the growing number of artificial structures^6,7^, eutrophication, fisheries and climate change^8–10^. The introduction of man-made structures can potentially have both positive and negative effects on marine ecosystems^11–14^. Soft-bottom communities are altered close to artificial structures^15–17^, while a significant amount of marine growth colonises the artificial hard structures^18,19^.

To assess the effects of man-made structures on the benthic community, most environmental impact assessment data collection studies have been conducted over small spatial and temporal scales^20^ such as single turbines or single OWFs and associated infrastructure^15,21,22^. Some countries have coordinated programmes to standardise data collection methods on soft sediments (e.g., Germany^23^, Belgium^24^, the Baltic Sea^25^), and there are existing methods to study macrofauna on natural hard substrates such as rocky bottoms^26^. However, there are no internationally agreed methods, metrics or databases for the data collection, which is critical for understanding the effects of artificial structures on marine ecosystems. Data are disparate owing to differences in data diversity, regarding (i) sampling devices and methods, (ii) sample analysis (e.g., variables, taxonomic resolution), (iii) data storage and management, as well as (iv) continuously changing taxonomy. This results in a lack of consistent data with regards to offshore artificial structures and benthos. Thus, investigation of large-scale benthic effects requires merging data from different sources, which is challenging (time consuming, costly, difficult) or even not possible^19^. Taken together, the available data are underutilised.

A few attempts have been made to collect and analyse biodiversity data from different substrates (wind turbines, oil and gas platforms, surrounding soft sediments and rocky reefs) in a single region^19,27,28^. Ecosystem-based management requires a deep understanding of the effects of artificial structures over large spatial and temporal scales that exceed budgets, timeframes and jurisdictional borders. Data sharing through the creation of an integrated database can provide multiple benefits for science, industry, and policy. It could be used for large-scale research studies examining the aforementioned effects and facilitate ecosystem-based management. Furthermore, the creation of a centralised dataset could enable answering scientific questions regarding stepping stone effects beyond the scale of individual OWFs, platforms or countries^29,30^. Industry could exploit this dataset for environment-friendly planning, predicting effects of new activities at offshore locations. Finally, sharing such data is crucial in developing fact-based scientific advice for decommissioning decisions for various stakeholders.

This paper presents the first data collection ‘Biodiversity Information of benthic Species at ARtificial structures’ (BISAR). BISAR contains data on benthic macrofauna collected in environmental impact studies, scientific projects and species inventories conducted at 17 artificial offshore structures in the North Sea between 2003 and 2019. The structures include OWFs, oil and gas platforms, a research platform and a geogenic reef to compare natural and artificial reef communities. BISAR includes data from soft and hard substrate studies (34 artificial structures), allowing comparisons of changes in both habitat types. This data collection currently contains data from a total of 3864 samples with 890 taxa. BISAR is the first data product containing harmonised and quality-checked international data on benthos from substrates influenced by artificial structures in the North Sea. Various stakeholders (e.g., industry, public authorities, research) will profit from the BISAR data collection as the greatest challenge in an era of blue growth is to get access to data from various sources^31^.

## Methods

BISAR contains data on a total of 3,864 samples taken at 1,453 stations with 68,433 records of 890 taxa of benthic macrofauna, from 34 artificial structures and surrounding soft bottom collected during 105 cruises between 2003 and 2019 (Table 1).

**Table 1:** A summary of the information compiled per individual datasets combined in the BISAR data collection, including dataset names, DOI links of data publications in repositories and related papers, country, location, water depth (ranges, m), years (ranges), structure type with number of artificial structures sampled in brackets, substrate sampled as well as the numbers of cruises, stations, samples, records, and taxa per Location.

Dataset	DOI (Data publications)	DOI (Related papers)	Country	Location	Water depths	Years	Structure type	Substrate type	Cruises	Stations	Samples	Records	Taxa
alpha ventus*	10.1594/PANGAEA.943528		DE	alpha ventus	1–36	2008–2012	OWF (4)	Soft & Hard	17	607	1461	30,766	418
Stukplus*	10.1594/PANGAEA.942727	10.1016/j.dib.2022.108790	DE	alpha ventus	27–37	2008–2011	OWF	Soft	4	176	668	12,045	184
Belwind, C-Power*	10.24417/bmdc.be:dataset:2680		BE	Belwind	15–30	2010–2019	OWF (3)	Hard	20	22	64	1,256	146
			BE	C-Power	4–30	2008–2019	OWF (3)	Hard	37	38	130	2,950	163
Borkum Reef		10.1016/j.seares.2015.06.010	NL	Borkum Reef	28	2013	GR	Hard	1	3	11	178	94
DanTysk, Horns Rev, Sandbank*	10.1594/PANGAEA.943480		DE	Dan Tysk	0–10	2018–2019	OWF (2)	Hard	2	12	36	963	111
			DE	Sandbank	0–10	2019	OWF (2)	Hard	1	6	18	371	67
			DK	Horns Rev	0–10	2003–2005	OWF (6)	Hard	6	109	793	8,100	96
FINO 1	10.1594/PANGAEA.805200		DE	FINO 1	1–30	2004–2007	RP (1)	Hard	11	218	218	2,580	129
Petrogas GBS		10.1016/j.seares.2020.101968	NL	Petrogas GBS	17–24	2019	OG (1)	Hard	1	1	39	1,104	114
Neptune, PAWF		10.1093/icesjms/fsy092	NL	Neptune	0–32	2014–2015	OG (8)	Hard	1	8	128	4,054	335
			NL	PAWF	0–25	2011–2013	OWF (4)	Soft & Hard	4	253	298	4,066	212
								**TOTALS**	**105**	**1,453**	**3,864**	**68,433**	**890**

Taxa number gives the absolute taxa number per Location defined in this table (e.g., taxa found in the Location alpha ventus). Please note that total taxa number does not sum up as taxa can occur in several datasets. Data unique to the BISAR collection (not published before) are marked with an asterisk. DE = Germany, BE = Belgium, DK = Denmark, NL = the Netherlands. OWF = offshore wind farm, OG = oil & gas rig, RP = research platform, GR = geogenic reef.

### Data compilation

**Fig. 1:**
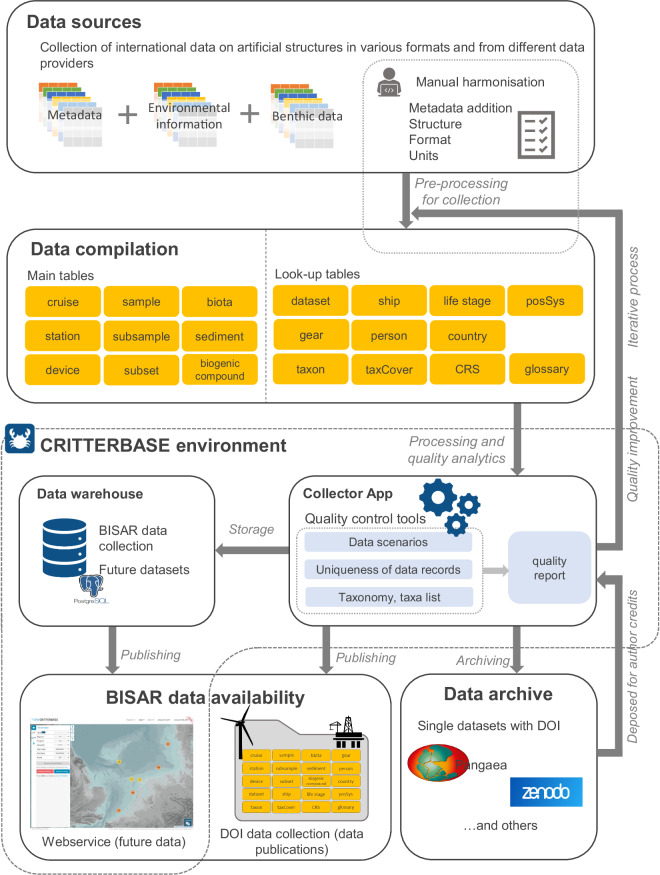
Workflow of the data compilation, quality check, storage and publication of the BISAR data collection.

The data were compiled from various sources, such as different international research institutes, universities, and industry. Hence, the format between different datasets varied and the number of metadata was highly variable. To harmonise the data, one data template was created and used by all data providers (Fig. 1, MSExcel-table) containing main tables and metadata information (lookup tables) (see section ‘data records’). The main tables contain metadata on the cruise, station, and individual samples (e.g., scrape samples on turbines) which were compiled for the first time in a harmonised style for the BISAR data collection. Biota data and environmental data (e.g., substrate of samples) were also compiled. Look-up tables collect additional information, e.g., sampling gear and life stages of biota. A glossary of the BISAR data collection provides detailed information about the data columns, as well as units of column data and a brief description.

Each dataset was individually transferred to the BISAR data collection by copying data columns from the original datasheets in the corresponding columns of the multi-sheet format. The compilation of the data was done between the data contributors and experienced data curators in an iterative process until the datasets were combined in the BISAR data collection (Fig. 1).

### Notes on benthic sampling

**Fig. 2:**
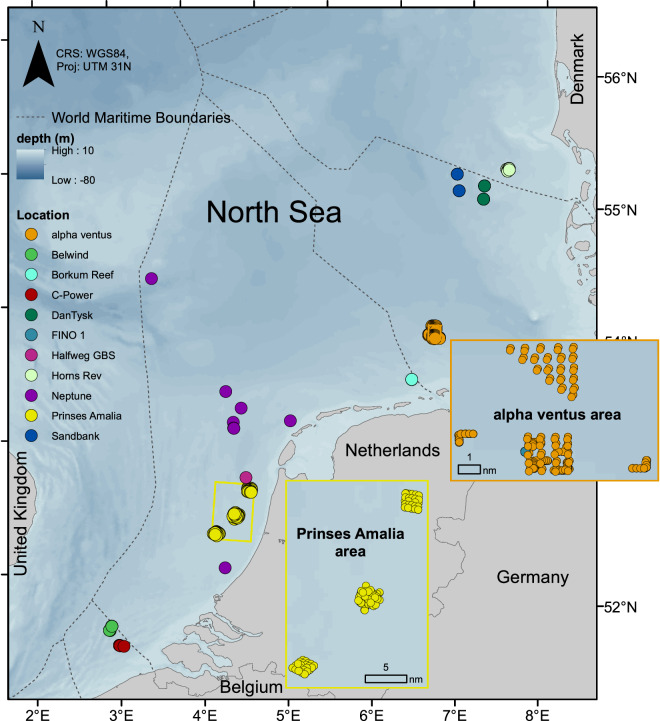
Map of sampling locations of the BISAR data collection. Alpha ventus and Prinses Amalia areas are displayed with zoom areas due to the large number of samples.

Benthos samples were collected within the different projects which included seven OWFs, nine oil and gas platform foundations, one research platform and one geogenic reef (Fig. 2) in the North Sea. Different methods were applied to collect samples from the hard substrates on and around the artificial structures. Specifics for each artificial structure or platform are described in the following sections.

#### FINO 1 research platform, Germany

The research platform FINO 1 is similar in size and shape to today’s jacket foundations of offshore wind turbines^32^. Sampling of the artificial hard substrate took place between April 2005 and October 2007 at nine different water depths between 1 and 28 m. At each depth, one to two samples were taken at random positions on vertical surfaces. Samples measuring 20 × 20 cm were scraped and captured in a mesh bag (mesh size: 0.5 mm) by scientific divers. Aboard all samples were preserved in 4% borax-buffered formalin. In the laboratory, specimens were pre-sorted, stored in 75% ethanol and then identified. Colonial species were counted as present, while individual species were counted and wet weighed. Wet masses were corrected by a factor of 1.2 to account for changes due to storage in ethanol (following Zintzen *et al*.^33,34^).

#### Alpha ventus, Sandbank and DanTysk offshore wind farms, Germany

At alpha ventus, both epifauna communities on the structures and seabed communities (wind farm area and reference area) were sampled. Sandbank and DanTysk were sampled for the fauna colonising the artificial hard substrate. All three datasets were collected according to the standard investigation concept for OWFs in the German part of the North Sea (latest version: StUK 4^23,35^).

Data from alpha ventus were collected in autumn 2009 and spring and autumn in the years 2010–2012. Sandbank was sampled in August 2019 and DanTysk in September 2018 and August 2019. Four turbines were investigated in alpha ventus (i.e., 2 jacket structures, 2 tripods), two turbines in Sandbank and DanTysk (all monopiles). Scrape samples with a surface area of 0.04 m² were collected by scientific divers at three or four different depths (alpha ventus at 1, 5, 10 and 15 m, Sandbank and DanTysk at 1, 5, 10 m), each with three replicates. Organisms were collected in mesh bags with a mesh size of 0.5 mm and conserved in 4% borax-buffered formalin. In the laboratory, taxa were identified, and individuals were counted and wet weighed.

Data from the project StUKplus Benthos also included seabed communities from alpha ventus only (in autumn 2008–2011)^36^. Samples were taken with Van Veen grabs (surface area 0.1 m²). The dataset served as a complementary investigation to the alpha ventus StUK monitoring, specifically to capture data relating to potential processes occurring at different distances from single wind turbines of the wind farm. Samples were collected at four sites, i.e., at two wind turbines inside the OWF alpha ventus and in two reference areas outside the OWF, with one transect in the main water current direction and one perpendicular to each other. Along each transect, seven stations, each 100 m apart (within OWF) and four sampling stations, each 200 m apart (within reference areas) were sampled. At each station, three replicate Van Veen grabs were sampled. Organisms were sieved over a 1 mm mesh and conserved in 4% borax-buffered formalin. Taxa were identified and individuals were counted and weighed (wet mass) afterwards in the laboratory.

#### Horns Rev I offshore wind farm, Denmark

In the Horns Rev I wind farm in Denmark, two sampling surveys of epifauna organisms per year were conducted on the monopile foundations and scour protection layer, during three years i.e., March and September of 2003, 2004 and 2005^37,38^. Scrape samples of 0.04 m^2^ were collected by scientific divers in the direction of the principal water current on both the upstream (SSW) and the downstream sides (NNE). Samples were taken at depths of 0, 2, 4, 6 and 8 m depth at the monopiles and from rocks in the scour protection layer at depths between 6 and 10 m. Two replicates per depth and three per rock were collected in bags with a mesh size of 1 mm. All samples were preserved in ethanol until further processing in the laboratory, where the samples were sorted under a stereomicroscope. All organisms were identified, counted and their ethanol wet mass was determined.

#### Prinses Amalia wind farm, the Netherlands

In the Prinses Amalia wind farm (PAWP), the fauna both on the structures and in the seabed were studied. In total, four turbine foundations were selected, both at edges and at the centre of the wind farm. Monitoring of the epifauna on the monopile foundations and scour protection layer was conducted three and a half (October 2011) and six (July 2013) years after construction^27,39,40^. Scrape samples taken by scientific divers were collected at five different depths: splash zone, 2, 5, 10 and 17 m depth from both sides of the turbine (SSW and NNE) with a single sample per depth-side combination. The sampled surface area was 28 × 20 cm (0.056 m^2^) and scraped fauna were collected in a sampling net (mesh size: 0.25 mm). The scour protection layer was sampled by collecting small rocks.

Soft-sediment fauna was collected within the wind farm in March 2012 and April 2013^39,40^ with 1 sample per station, from the wind farm and reference areas south-west and north-east of the wind farm. Soft sediment fauna was sampled using a box corer grabbing an area of 0.078 m^2^. The samples were sieved on board on a sieving table with mesh size of 1 mm.

All samples and rocks were fixed with a borax buffered 5% formaldehyde solution. In the laboratory, all organisms were identified, counted and their biomass (both wet and ash-free dry mass) was measured. Colonial species were noted as present per sample, while their biomass was not determined.

#### Borkum Reef grounds, oil and gas platforms Neptune Energy & Petrogas GBS, the Netherlands

The Borkum Reef Grounds is an area with geogenic reefs at the border between the Netherlands and Germany. Neptune and Petrogas are oil and gas platforms in the Netherlands. The fauna of the geogenic reefs and large hard substrates from platforms were sampled by scientific divers by scraping, using an airlift sampler with nets of mesh size 0.5 mm^27,28,41^. Sample size was 0.05 m^2^. On the scour protection layer around the Neptune platforms, individual rocks or gravel were collected from an area of 0.05 m^2^. Around the Petrogas Gravity Based structure (GBS), large rocks were sampled by scraping fauna from within a 0.05 m^2^ frame and collecting it in an airlift sampler with nets of mesh size 0.5 mm. On board, samples were rinsed from the nets and conserved on ethanol 99% or borax buffered formaldehyde 6%. Rocks with epifauna were conserved whole without sieving. In the laboratory, all taxa were identified, counted and weighed (all wet, some also ash free dry mass). Colonial species surface area covered was estimated to the nearest cm^2^, but the species were not weighed.

#### Belwind and C-Power offshore wind farms, Belgium

The long-term monitoring of Belgian offshore wind farms started in 2008^42^ and is still ongoing. The monitoring data presented here refer to monopile foundations from the Belwind OWF (since 2010), and jacket and gravity-based foundations from the C-Power park (since 2008). During the first two years, the sampling occurred seasonally (spring, summer, autumn) and across various depths of the selected foundations. Later, the samples were collected once a year in summer or autumn, at approximately 15 m depth and from the scour protection layers. In Belwind, four monopile foundations were sampled throughout the monitoring period, but not simultaneously. In C-Power, the gravity-based turbine D5 was sampled throughout the entire duration of the monitoring, except in 2016 when samples were taken from D6. The jacket foundation from C-Power was sampled only once (one year after installation). The sample size of the scrape samples taken by scientific divers was 0.0625 m^2^ and scraped material was collected in a plastic zip lock bag. Scour protection layer stones were collected manually by divers and were brought to the surface in plastic sample bags. All samples were preserved in buffered 4% formalin. In the laboratory, the samples were sieved using a 1 mm mesh size sieve and organisms were sorted and identified. Individual species were counted while for colonial species only presence was scored.

**Fig. 3:**
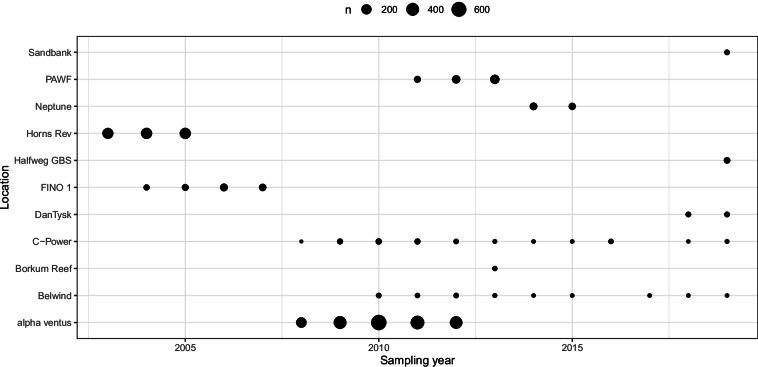
Number of samples taken per location and year.

**Fig. 4:**
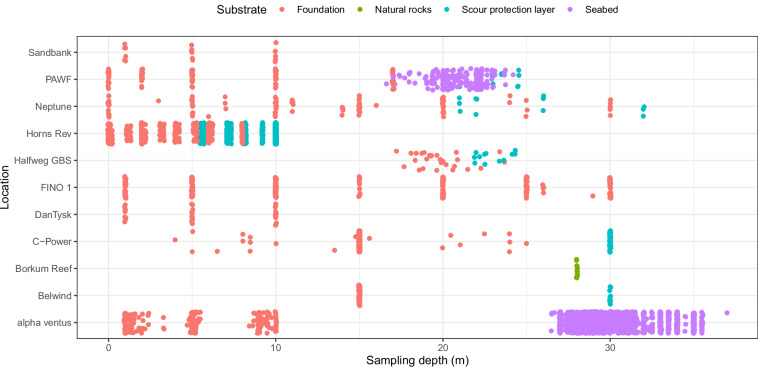
Substrate sampled per location and depth. To illustrate variations in the number of samples and variations in substrates at locations at the same depth, a small amount of random noise (jitter) was added to the positioning of the points in the x and y direction. To illustrate variations in the number of samples and variations in substrates at locations at the same depth, a small amount of random noise (jitter) was added to the positioning of the points in the x and y direction.

Due to the different purposes of the projects from which the data originate, and the various construction periods of the artificial structures, there is variation in the number of samples and the timing of sampling and water and sediment depths at which the samples were taken. The total number of samples per location varied from 11 (Borkum Reef) to 1,984 (alpha ventus; Fig. 3). Sampled water depths varied between 0 and 37 m, and some structures were sampled throughout the water column (e.g., FINO 1, Neptune, Horns Rev), while at some locations only the top half of the water column was sampled (e.g., alpha ventus; DanTysk, Sandbank; Fig. 4).

BISAR is a valuable dataset to investigate the colonising epifauna on different artificial structures and the benthos surrounding the structures. Descriptions and details about the study designs, including reference sites for the different artificial structures, are given in Table 1 (see column DOI (Related papers)). The currently unpublished BISAR collection has already been used in scientific studies e.g., to estimate the effects of decommissioning of offshore wind farms on benthos^43^; to investigate long-term cumulative impacts of offshore wind farms on biodiversity^44^; and some of the data have been applied to compare biodiversity on platforms, wind farms and rocky reefs^27^ and in the analysis of generalised benthic changes after wind-farm construction^19^. These published studies highlight the potential application of the BISAR data collection.

## Data Records

The BISAR data collection is available at figshare^45^ as part of this publication. The data collection is composed of 11 tables as multi-sheet format (*.xlsx format) and contains primary data and meta-data structured in main tables and lookup tables for further information (Fig. 1). The first table contains a detailed glossary with information on all tables, headers, and units of the data. The following tables and contents are included in the file in separate tabs:

Main tables:

- Cruise - Data on sampling cruises, including vessel names, dates and region visited.
- Station - Station coding for stations visited in cruises, dates, coordinates and local depth.
- Sample - Samples collected at each station, with data on gear type used, sampled area and coordinates and depths of each sample. Here, information on artificial structures, data on the platform type, foundation type, construction material and sample type were included.
- Biota - Information on the species observed in the samples, with species name, data on the AphiaID as used in WoRMS^42^ (World Register of Marine Species), number of individuals (i.e., counts of individuals) and presence/absence data, weights (wet, dry or ash free dry mass in g) and fraction of sub sample.
- Sediment - Sediment characterisation data on samples, with grain sizes, sample weights and loss of mass on ignition.
  Lookup tables:
- Dataset - Information on the datasets and projects that were included in BISAR, including a DOI and short project description.
- Gear - All gear types used to collect the samples included in BISAR.
- Taxon - All unique taxa included in BISAR, with WoRMS export data.
- Person - Names, affiliation and contact information of the scientists leading the projects on which BISAR is based.
- Lifestage - Different ontogenetic life stages of the species included in the biota sheet, e.g., remarks on whether a species is juvenile, larval, etc.
- CRS - Coordinate Reference System on which the positions of each station and sample are based.

Summary information for each location included in BISAR is provided in Table 1. All datasets are published, some as a supplement to a publication (secondary source), others as data publications (i.e., DOI data publication) (see Table 1).

## Technical Validation

This section describes the procedure on how the single datasets from various sources were quality checked and harmonised to combine all data in the BISAR data collection. One of the greatest challenges during the BISAR data compilation was dealing with multiple methods and sampling devices, and the fact that the basic entity of record, i.e., the taxonomy of a species, is subject to change. Taxonomic records of species can change over time as taxa are re-described and given synonyms.

Harmonisation of different datasets requires knowledge of the data. Thus, the compilation, harmonisation and quality control were carried out in an iterative process between data providers and curators (see iterative process, Fig. 1). This process was carried out in several rounds until the datasets could finally be included in the BISAR data collection.

During this iterative ingest, we applied several levels of quality checks. There are two different data scenarios in the compiled dataset: biotic data are provided either as numbers (i.e., counts) or as presence/absence data. Wet mass is only optionally provided and is not available for all datasets. The counts refer to different sampling surface areas from e.g., scrape samples or Van Veen grabs. The applied quality routines compare the given “sampled surface area” with the area of the sampling gear to ensure that count data are set in relation to an area for future standardisation. In addition, we checked the uniqueness of the following quadruple per sample (i.e., per smallest entity of the data collection): (i) AphiaID, i.e., a taxon-specific ID provided by WoRMS^46^, (ii) life stage (e.g., juvenile), (iii) sieve mesh size, and (iv) taxonomic specification (e.g., cf., indet.). The same AphiaID may be named more than once in the same sample only if it differs in its combination with the three other attributes. Finally, the taxonomy of taxa was controlled by several steps within the data compilation process: (a) taxonomy was synchronised by queries to WoRMS with the respective AphiaID, (b) missing taxa information was added (e.g. taxonomic tree and relatedness of species) and (c) a comprehensive taxa list across the respective datasets was added to the BISAR data collection. This ensures a harmonised taxonomy for all datasets. All these quality component activities lead to repeated compilation of datasets until final inclusion in the BISAR data collection.

Additional data quality checks during the iterative compilation process of BISAR data collection were conducted using the CRITTERBASE Collector App (Fig. 1)^47,48^. CRITTERBASE is a biodiversity information system hosted by the Alfred Wegener Institute that can handle sample-based and organism related marine data. For a full description of data quality control, see the publication^44^ and https://critterbase.awi.de/#qc. Our data quality check showed the high-quality harmonisation of international data required an interdisciplinary team of biologists, data and computer scientists as harmonised metadata and high-quality plausibility checks on the reliability of raw data. Therefore, it is recommended that technical and biological expertise is considered across these types of exercises.

## Usage Notes

Single data sets may be downloaded from published sources listed in Table 1. The complete BISAR data compilation is published as part of this publication^45^ (Fig. 1). Further, BISAR data compilation will be available via the CRITTERBASE web portal https://critterbase.awi.de/bisar. Therefore, the entire data collection or excerpts could be downloaded (Table 1). New data additions from ongoing monitoring and new locations will be available by the same web portal in the future.

BISAR includes quality-checked raw and original benthic data from various sources and artificial structure types. Please note that taxa of the BISAR collection were checked for synonyms and actual taxon names via WoRMS during the quality checks. However, biotic data contain the original name (from laboratory analysis), while the valid taxon names (via WoRMS) are provided in the species reference table of BISAR. In addition, the data originate from different laboratories and taxonomists with different degrees of taxonomic precision. For example, the anemone *Metridium senile* may be reported with different names, i.e., ‘*Metridium senile’*, ‘*Metridium*’, ‘*Anthozoa*’, in the BISAR data collection. As with all benthic raw data, taxonomy must be further harmonised for the user’s purposes, e.g., by combining taxon entries to higher taxonomic levels, as computable analysis corresponds to several taxa. Thus, working with the BISAR data collection needs some level of benthic, taxonomic knowledge.

Counts of individuals and presence/absence data are given in different columns in the BISAR data collection, and may need to be combined for further analysis. The count data refer to different “sampled surface areas” of the different sampling gears (i.e., grab, scrape samples with different sampling areas). The “sampled surface areas” are provided for all samples in BISAR. Thus, users must consider sample size and “sampled surface areas” to standardise count data (e.g., individuals per m^2^) for the user’s purposes.

The BISAR data compilation summarises the raw data from different projects as our intention was to provide as much details and data from the different studies as possible. Thus, the data are not homogenised, i.e., different sieve mesh sizes and sampling gears. Instead, we provide all necessary information to homogenise the data by the users themselves. We therefore suggest that users working with this data compilation familiarise themselves with the detailed information in the metadata, look-up tables, and glossary of data, which, when combined with benthic knowledge, will help them handle the data properly.

## Data Availability

The source code for the Collector App for CRITTERBASE - the software for data quality checking and storage - is available as a free download under the open-source BSD 3 licence (10.5281/zenodo.5724020)^48^. The software libraries and versions used are referenced in the README.MD file.
